# Measles Transmission at a Domestic Terminal Gate in an International Airport — United States, January 2014

**Published:** 2014-12-19

**Authors:** Jared S. Vega, Miguel Escobedo, Cynthia R. Schulte, Jennifer B. Rosen, Stephanie Schauer, Rachel Wiseman, Susan A. Lippold, Joanna J. Regan

**Affiliations:** 1 Division of Global Migration and Quarantine, National Center for Emerging and Zoonotic Infectious Diseases, CDC; 2 New York State Department of Health; 3 New York City Department of Health and Mental Hygiene; 4 Wisconsin Department of Health Services; 5 Texas Department of State Health Services

In March 2014, CDC identified a possible cluster of four laboratory-confirmed measles cases among passengers transiting a domestic terminal in a U.S. international airport. Through epidemiologic assessments conducted by multiple health departments and investigation of flight itineraries by CDC, all four patients were linked to the same terminal gate during a 4-hour period on January 17, 2014. Patient 1, an unvaccinated man aged 21 years with rash onset February 1, traveled on two domestic flights on January 17 and 18 that connected at the international airport. Patient 2, an unvaccinated man aged 49 years with rash onset February 1, traveled from the airport on January 17. Patient 3, an unvaccinated man aged 19 years with rash onset January 30, traveled domestically with at least a 4-hour layover at the airport on January 17. Patient 4, an unvaccinated man aged 63 years with rash onset February 5, traveled on a flight to the airport on January 17.

Patients 1 and 2 traveled on the same flight from the airport and were seated one row apart; both spent time at the departure gate before the flight. Patient 3, whose flight departed after the flight of patients 1 and 2, also reported spending time at this gate area during the time that patients 1 and 2 were present. Patient 4 passed through the same domestic gate around the time the other three patients were waiting to depart.

For cases in three of the patients genotyping was performed and identified the measles strain as B3, the predominant strain circulating in the Philippines and in the United States in early 2014 (1). Based on the available information, it is likely that transmission occurred in the airport at the domestic gate. The source case of this presumed cluster was not identified, and no other cases were identified beyond this cluster of four cases.

Measles transmission has occurred in airports, an environment in which travelers from measles-endemic areas or areas where outbreaks are occurring are likely to be present (2,3). The exposures in this report were not prolonged and occurred in a domestic rather than an international terminal, highlighting the fact that measles is highly contagious and that measles continues to pose a risk for infection among unvaccinated persons in the United States. Ensuring that all susceptible travelers are vaccinated against measles is an important way to decrease the spread and importation of measles in the United States (1). Airports and other travel venues should be considered as potential exposure settings when investigating cases.
